# Nuclear Receptor Variants in Liver Disease

**DOI:** 10.1155/2012/934707

**Published:** 2011-12-21

**Authors:** Roman Müllenbach, Susanne N. Weber, Frank Lammert

**Affiliations:** ^1^Department of Medicine II, Saarland University Medical Center, 66421 Homburg, Germany; ^2^Molecular Hepatology, Alcohol Associated Diseases, II. Medical Clinic Mannheim, 68167 Mannheim, Germany

## Abstract

This review aims to provide a snapshot of the actual state of knowledge on genetic variants of nuclear receptors (NR) involved in regulating important aspects of liver metabolism. It recapitulates recent evidence for the application of NR in genetic diagnosis of monogenic (“Mendelian”) liver disease and their use in clinical diagnosis. Genetic analysis of multifactorial liver diseases such as viral hepatitis or fatty liver disease identifies key players in disease predisposition and progression. Evidence from these analyses points towards a role of NR polymorphisms in common diseases, linking regulatory networks to complex and variable phenotypes. The new insights into NR variants also offer perspectives and cautionary advice for their use as handles towards diagnosis and treatment.

## 1. Introduction

Systematically, genetic analysis with regard to disease onset and progression can be separated into pre- and post-hoc examination of monogenic or polygenic diseases. Monogenic (“Mendelian”) diseases are caused by a single gene defect and follow relatively straightforward inheritance patterns. The most prominent of these disorders are rather rare, often severe, and characterized by early onset. Genetic testing for monogenic liver disease in symptomatic patients is based on known disease-associated gene variants, thereby confirming the genetic etiology and sometimes allowing prediction of disease progression [1].

 In contrast, polygenic diseases such as fatty liver disease and gallstones result from combinations of multiple gene variants and environmental factors, all of which play a role in disease initiation and progression [2]. The assessment of predisposition towards polygenic disease is based on sequence analysis of known contributory genes and construction of “polygenic risk scores” from variants of these genes [1]. Still in its infancy, personal genome information might eventually be able to predict a variety of risks associated with an individual's lifestyle such as fatty food and alcohol consumption, as well as susceptibility to infectious diseases such as infection with hepatitis B or C virus.

## 2. Nuclear Receptors

**Figure 1:**
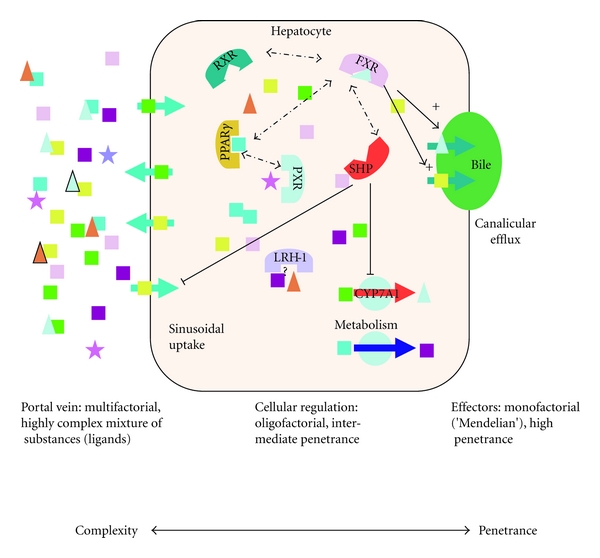
Schematic depiction of NR action in hepatocytes demonstrating a reduction in complexity and an increase in penetrance of genetic variants from the sinus to the canaliculus. Squares represent metabolic compounds such as triglycerides, cholesterol, fatty acids, and phospholipids; triangles represent bile salts; stars represent toxins; large semi-circles symbolise nuclear receptors, and circles stand for metabolic enzymes.

Nuclear receptors (NRs) are a subclass of regulatory molecules that orchestrate gene transcription in response to the presence or absence of specific ligands. Due to these functional requirements, they are characterized by the presence of a ligand-binding and a DNA-binding domain. NRs represent a central point of interaction between environment and gene regulation. They are the “hinge” connecting endogenous and environmental stimuli, that is, ligands, with the cells' transcriptional response (Figure 1).

 This position makes them a prime target for medical intervention by agonistic or antagonistic binding of synthetic compounds. However, the regulatory orchestra of molecules conducted by NR is highly complex and abounds with redundancy and crosstalk, hence any impact might be potentially difficult to predict as well as disappointingly diffuse. Even identical variations in a single NR can result in a wide variety of phenotypes due to genetic differences in the cofactors involved and higher order networks based on mutual regulatory interaction [3].

 The core position in maintaining cellular equilibrium should render NR susceptible to the impact of natural sequence variation. On the other hand, it has been suggested that the phenotypic effects of NR gene variation are bound to be less severe than a variation in functionally defined effector molecules such as a single-substrate transmembrane transporter. The transcriptional regulation directed by NR is kept in tight check and fine-tuned by a set of co-regulators (co-activators or corepressors) [4, 5]. Consequently, relatively few congenital, “Mendelian” diseases have been identified to date, which are caused by genetic variations in NR. One example is maturity-onset diabetes of the young (MODY1), caused by mutations affecting the gene encoding the hepatic nuclear factor (HNF) 4-alpha (*NR2A1*) [6] (Table 1).

 While the denominator of “monogenic disease” seems to imply a straightforward genotype-to-phenotype correlation, the reality in even those seemingly “simple” diseases is anything but simple: Tirona et al. [7] showed that HNF4A is critically involved in the PXR (NR1I2)- and CAR (NR1I3)-mediated transcriptional activation of cytochrome P450 (CYP) 3A4, hence involving two more NRs even in its basal regulatory function. Whereas Hani et al. [8] identified *HNF4A* mutations as being causative in maturity-onset diabetes of the young (MODY type 1, OMIM #125850) based on a nonsense mutation (p.Q268X) in an extended pedigree, many other seemingly functional variants have turned out to be either innocent bystanders or of relatively low penetrance. Pearson et al. [9] were able to show that functional *HNF4A* variants are associated with a considerable increase in birth weight and macrosomia, and a novel cause of neonatal hypoglycemia. Results from the investigation of 108 members of 15 families with MODY1 show how genotype-phenotype correlation is far from clearcut, with both described phenotypes being found only in 15–56% of mutation carriers. Ek et al. [10] examined the impact of two disease-associated variants (p.T130I and p.V255M, Table 1) on various aspects of HNF4-alpha function. Both variants showed decreased transactivation. Only the p.T130I polymorphism was associated with T2D, whereas the p.V255M variant was associated with a decrease in fasting serum C-peptide levels. Array analyses revealed that HNF4-alpha bound to the promoters of 12% of hepatocyte islet genes represented on a microarray and hence can be considered a “master regulator” of hepatocyte and beta-cell genes [11]. But even in a complex and occasionally ambiguous setting, where the detection of a functional variant does not necessarily predict disease phenotypes, genetic testing appears to be helpful, if only to identify at-risk relatives and motivate affected individuals towards lifestyle changes [12]. Evidently, there is no clear delineation between the “Mendelian” diseases and contribution towards complex phenotypes modulated by NR variants (Figure 1). A meta-analysis of polymorphisms in the promoter and along the entire coding region of the *HNF4A* gene and type 2 diabetes in 49.577 individuals revealed significant associations for more than one locus [13].

 Nuclear transcription factors are known to undergo posttranslational modifications modulating their regulatory activity, which obviously makes the interpretation of genetic tests more difficult. Recent findings of epigenetic modification of the *HNF4A* promoter add an additional layer of uncertainty and environmental impact. Maternal diet and aging alter the epigenetic control of a promoter-enhancer interaction at the *Hnf4a* gene in rat pancreatic islets [14]. Environmentally induced changes in promoter-enhancer interactions might represent a key epigenetic mechanism by which nutrition can influence NR signaling.

## 3. Candidate Receptor Studies

### 3.1. Vitamin D Receptor (VDR/NR1I1)

The human vitamin D receptor (VDR/NR1I1) has been in the focus of research for over a decade, a main reason being a wide spectrum of known effects of vitamin D deficiency. A second, more mundane reason might be the availability of four frequent variants (rs7975232, *Apa*I; rs1544410,* Bsm*I; rs10735810, *Fok*I; rs731236, *Taq*I) amenable to relatively quick and easy analysis using simple technology that has been available in every genetics laboratory, restriction fragment length polymorphism (RFLP) analysis. A Pubmed search (as of June 7, 2011) on “vitamin D receptor polymorphism” resulted in 1,200 articles, covering a wide-range of associated biochemical processes and diseases ranging from the more obvious bone density in various species at various ages [15, 16] to Parkinson disease [17] within the first 20 hits, ulcerative colitis [18], and inflammatory bowel disease [19]. Limiting the search to the liver results in a more manageable set of less than 30 publications, with a detectable focus on inflammatory and autoimmune liver diseases, in particular autoimmune hepatitis and primary biliary cirrhosis [20–22], but also hepatitis B virus (HBV) infection [23] (Table 1).

 In contrast to the mouse liver, which showed no VDR expression [3], VDR was detected mainly in the nonparenchymal cells of rat liver, whereas hepatocytes expressed barely any VDR in murine livers [24]. Human hepatocytes express VDR, albeit at very low abundance [25]. One of the potential ligands of hepatic VDR in humans is the secondary bile acid lithocholic acid, resulting in a repression of bile salt synthesis by transcriptional repression of cholesterol 7*α*-hydroxylase (CYP7A1), the rate-limiting enzyme in bile salt biosynthesis [26]. The effect is achieved by competing for promoter binding with HNF4-alpha. This example shows that results from animal models have to be treated with caution, and once again illustrates the complex interaction between NR-regulated pathways in human liver.

 Association studies between gene variants and diseases provide signposts towards genes underlying functional effects, but do not elucidate how these effects are achieved. Investigations into the detailed effects of the respective polymorphisms in NR are harder to interpret than similar investigations in other molecules of clearer functional delineation [27]. Cell-type specific splicing events might modulate transcriptional activation or ligand binding and cause effects in a substrate-dependent manner. As an example, a functional effect of the 3′*Bsm*I polymorphism in intron 8 of the *VDR*/*NR1I1* gene was shown to have a modulatory function on epithelial cell proliferation when combined with the effects of calcium [28]. The *Fok*I polymorphism of *VDR/NR1I1* results in distinct translation initiation sites and was shown to have an effect on cell growth inhibition, possibly through estrogen receptor-*α* protein repression in a cancer cell line [29]. These pleiotropic and highly variable functions go some way towards explaining the miscellany of associations that have been detected for *VDR* polymorphisms, among others with the occurrence of hepatocellular carcinoma (HCC) in patients with liver cirrhosis, particularly in patients with an alcoholic etiology [30]. They also show why the idea of using NR as a handle towards personalized treatment of patients is not straightforward, due to the high number of unspecific side effects.

### 3.2. FXR: The Central Bile Salt Sensor

**Figure 2:**
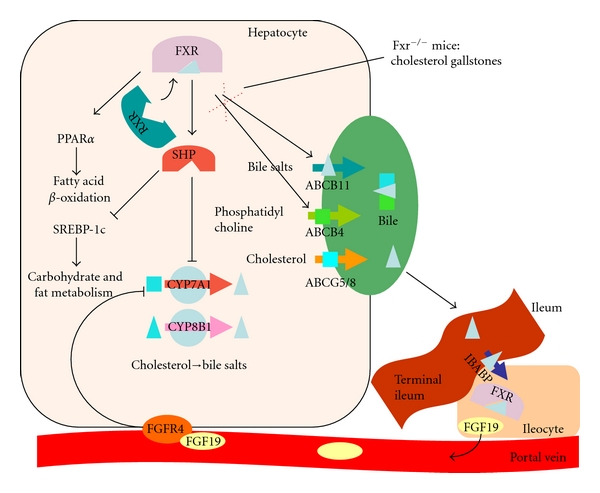
Schematic diagramme showing various aspects of FXR function as an example of complex nuclear receptor regulation and interaction. IBABP: intestinal bile acid binding protein, SREBP-1c: sterol regulatory element binding protein 1c, FGF: fibroblast growth factor, and FGFR: fibroblast growth factor receptor.

FXR/NR1H4 is the hepatic nuclear bile salt receptor, regulating bile salt synthesis and transport in hepatocytes, the central hub of cholesterol synthesis and conversion. Bile salts are direct FXR ligands and bind to the ligand binding domain of the molecule at low concentrations as dimers with the retinoid X receptor (RXR/NR2B1). Upon binding of the heterodimer, conformational change causes FXR activation. FXR also controls enterohepatic circulation through regulation of intestinal bile salt uptake via expression of the intestinal bile acid binding protein (I-BABP) in enterocytes. At the same time, FXR increases expression and release of fibroblast growth factor 19 (FGF19, mouse orthologue FGF15), which provides a feedback regulation loop from the intestine to the liver via association with *β*-klotho and activation of its dedicated receptor FGFR4 (Figure 2). In the liver, dimerization with RXR induces the expression of various genes involved in bile salt transport from the hepatocyte into the bile canaliculus such as the phosphatidylcholine floppase *ABCB4* and the bile salt export pump *ABCB11*. Interaction of FXR with the short heterodimer partner (SHP/NR0B2) decreases bile salt synthesis by repression of CYP7A1 and CYP8B1. Hence, FXR occupies a key role and is a prime target for manipulating the balance of bile salts in multiple parts of the enterohepatic circulation. However, results from a pilot experiment assessing the metabolic impact of a synthetic FXR agonist indicate that caution is warranted. The administration of GW4064-induced obesity and diabetes in mice fed a high-fat diet and worsened the metabolic effects in liver and adipose tissue [31].

 A potential role of FXR dysregulation in gallstone formation could be shown in the FXR deficient mouse. Due to the lack of positive feedback via FXR, the hepatocanalicular transporters *ABCB4* and *ABCB11* are not induced by bile salts (Figure 2) [32]. Under normal circumstances, biliary cholesterol is solubilized in mixed micelles, consisting of cholesterol, phosphatidylcholine, and bile salts. Lack of the latter two constituents causes supersaturation of cholesterol and the precipitation of crystals in the FXR-knockout mouse. When administered to gallstone-susceptible wild-type mice, the FXR agonist GW43456 reinstated the biliary balance of cholesterol, phospholipids, and bile salts by induction of hepatocanalicular transporter expression [32]. This makes FXR a potential target for the treatment of cholesterol gallstones.

#### 3.2.1. FXR Variation and Functional Conservation

A survey of genetic variation in 13 NR that control the expression of drug metabolizing enzymes revealed an intriguing paucity of known functional variants in the coding region of *FXR/NR1H4*, comparable only in numbers to the androgen receptor (*AR/NR3C4*) and the aryl hydrocarbon receptor (*AHR*) [33]. This scarcity has been speculated to be indicative of considerable evolutionary selective pressures that conserve the functional domains in these receptors. However, compared to the both aforementioned receptors with low frequency of functional polymorphisms in the coding region, *FXR* revealed a relatively high number of base substitutions in the regulatory sequence. Thus, protein abundance of the molecule appears to be more variable than its conformation. Comparison of the frequency of variants in the sequences of drug metabolizing CYP enzymes with the frequency in essential enzymes in protein biosynthesis (ribosomal genes) and NR genes revealed similar patterns. A high level of variation in the regulatory sequence and a high conservation in coding areas of NR genes was juxtaposed by a reverse distribution in ribosomal genes [33]. No difference in variation frequency was observed in the noncoding, intronic areas.

#### 3.2.2. FXR Variation in Complex Disease

Quantitative trait locus mapping in inbred mice identified the *Nr1h4* gene encoding murine Fxr as a candidate gene for a gallstone susceptibility (lithogenic) locus (*Lith7*). Sequencing, genotyping, and haplotype analysis in humans revealed no more than three frequent haplotypes accounting for >95% of the variability. Kovacs et al. [34] described an association of a common risk haplotype *NR1H4_1* (−20,647T; −1G; IVS7-31A) with gallstones in Mexican patients (OR = 2.1, *P* = 0.02) (Table 1). The association was inconsistent between different populations, pointing to a minor contributory role of FXR in overall gallstone susceptibility. Sequence variants of *FXR* have also been investigated in other pathological liver conditions, such as intrahepatic cholestasis of pregnancy (ICP). ICP is an interesting model disease as it illustrates the step up in complexity from monogenic diseases like severe familial cholestasis in children to complex cholestatic syndromes. The central role of FXR in balancing bile salt concentrations throughout the enterohepatic circulation makes it a good candidate for investigations into the causes of bile salt imbalances during pregnancy. Van Mil et al. [35] used an elegant and convincing experimental setup to prove the molecular impact of the few variants found in or near the transcribed sequence of *FXR*: They could show how two disease-associated single nucleotide polymorphisms (SNPs) at the methionine start codon or its immediate vicinity resulted in decreased translation. Expanding beyond the mere quantitative change, van Mil et al. [35] went on and proved that decreased translation diminished or abolished transactivation of FXR target genes in response to bile salt stimulation. A similar effect could be shown for the only nonconservative sequence variant that was found in both ICP patients and unaffected controls [35] (Table 1). Marzolini et al. [36] were able to confirm an effect of this variant *in vivo* by examining samples from a human liver bank. The expression levels of the *FXR* target genes short heterodimer partner (*SHP*/*NR0B2*) and organic anion transporting polypeptide 1B3 (*OATP1B3*) were reduced in livers harboring the rare [T] allele at position −1 of the *FXR*-coding sequence.

### 3.3. PPAR-Gamma and Diabetes

The peroxisome proliferator-activated receptor gamma (PPARG/NR1C3) is a fatty acid-activated member of the PPAR subfamily of NR. These receptors play important roles in lipid and glucose metabolism. Members of the family have been implicated in obesity-related metabolic diseases such as hyperlipidemia, insulin resistance, and coronary artery disease. Like FXR, PPARs form heterodimers with RXR, and these heterodimers regulate the transcription of various genes in liver. Polymorphisms in *PPARG*, particularly the proline-to-alanine substitution at aminoacid position 12, have been associated with diabetes, insulin levels, insulin sensitivity, body mass index, and dyslipidemia [37–40] (Table 1).

 Disease prediction for population subgroups based on a combined “diabetes risk matrix” including *PPARG* p.P12A has been proposed to be informative and might, if accompanied by lifestyle intervention, prove a worthwhile path for prevention [41, 42]. Genome-wide association studies (GWAS) of type 2 diabetes in 2,335 Finns confirmed a contributory role of *PPARG* [43]. In a comprehensive example of complementarity between human and animal model research, Heikkinen et al. [44] were able to show that the p.P12A variant exerts its impact on various aspects of metabolism and human longevity in a diet-dependent manner. Hence, this prominent member of the NR family of molecules is a leading example of gene × environment interaction and “nature via nurture”.

### 3.4. PPAR-Gamma and NAFLD

Meirhaeghe et al. [45] described an association of a silent SNP in exon 6 of the *PPARG *gene (c.C161T) and the level of circulating leptin in obesity. Obese subjects carrying at least one [T] allele displayed higher plasma leptin levels than homozygous carriers of the common allele. The [T] allele was also associated with lower BMI at a given leptin level, indicating a complex interaction between PPAR-gamma and leptin signaling. These findings could be confirmed and extended by a study in 96 Chinese patients with nonalcoholic fatty liver disease (NAFLD), which reported an association of this variant with adiponectin levels and the development of NAFLD [46]. Zhou et al. [47] replicated this finding in an independent cohort, demonstrating that *PPARG* c.C161T and other polymorphisms are associated with the levels of tumor necrosis factor (TNF)-alpha, leptin, and adiponectin in NAFLD. When patients in Germany (NAFLD and AFLD, *n* = 363) [48] and Italy (NAFLD, *n* = 202) [49] were analyzed for the p.P12A variant, the results were less conclusive: German patients with fatty liver disease of either etiology were more likely to carry the rare minor allele, but no association was detected between p.P12A and the severity of steatosis, necroinflammation, or fibrosis.

### 3.5. Interactions between FXR and PPAR-Gamma

Bile salts are intimately entwined with lipid metabolism, and besides their well-known role in dietary lipid absorption and cholesterol homeostasis, evidence is accumulating that the body uses blood levels of bile salts as sensor for metabolic processes (reviewed comprehensively by Thomas et al. [50]). Hence, bile salts have been considered as metabolic signaling molecules. The decrease in energy expenditure following reduction of the bile salt pool by treatment of mice with the FXR-agonist GW4064 is proof of this concept, although the resulting weight gain and insulin resistance are not a desirable outcome [31]. The observation that PPAR-gamma might be induced by FXR in hepatic stellate cells underscores and highlights the complex interaction between bile salts, metabolism, inflammation, and fibrogenesis [51]. It also adds weight to the argument that NRs are involved in most aspects of metabolic regulation and liver cells' response to both internal and external stimuli. Further complexity is added to the spectrum of NR interactions in the liver by a recent report on the effect of GW4064 on the expression of the PPAR-gamma coactivator-1alpha (PGC1*α*/*PPARGC1*). The synthetic agonist GW4064 enhances expression of PGC1*α* and thus mitochondrial function, however enhanced expression of FXR increases *PPARGC1* RNA not directly, but via protection from repression by the atypical corepressor SHP [52].

### 3.6. PXR and NAFLD

The pregnane X receptor (PXR/NR1I2) is known to be involved in the regulation of hepatic detoxification processes. Using PXR knockout and humanized mouse models, PXR was found to influence drug × drug interactions, hepatic steatosis, and the homeostasis of vitamin D, bile salts, and steroid hormones [53, 54]. Investigations into the genetic contribution of the *PXR* locus in 188 patients with NAFLD showed an association of two variants (rs7643645 and rs2461823) with several phenotypes of the disease, among others ALT levels [54] (Table 1). The combined analysis of both loci provided information with regards to disease progression. One of the associated SNPs (rs7643645) is localized within a potential binding site for HNF4-alpha, once again illustrating the interactions between NR pathways.

### 3.7. NR and Liver Cancer

Liver receptor homologue 1 (LRH-1, NR5A2) is an orphan member of the NR superfamily, that is, it has no known endogenous ligand. LRH-1 is involved in the regulation of genes that participate in steroid, bile salt, and cholesterol homeostasis [55]. Knockout of *Nr5a2* in mice results in compromised intestinal lipid absorption as well as defective embryogenesis and cholesterol homeostasis [56, 57]. Application of dilauroyl-phosphatidylcholine, a specific LRH-1 agonist, increased bile salt levels and lowered hepatic triglyceride and serum glucose concentrations [58]. In mouse models of type 2 diabetes, the LRH-1 agonist also decreased hepatic steatosis and improved glucose homeostasis, pointing towards a new intervention target for the treatment of type 2 diabetes. LRH-1 has also been implicated in the growth of liver tumors via reversal of repression by SHP: *In vitro* methylation of the *SHP* promoter reversibly decreased transactivation and LRH-1 binding; overexpression of *SHP* inhibited HCC foci formation, arrested HCC tumor growth in xenografted nude mice, and increased the sensitivity of HCC cells to apoptotic stimuli [59].

### 3.8. NR and Viral Hepatitis

The replication of hepatitis C virus (HCV) is linked to lipid droplets, and a combined genomic/metabolomic analysis of HCV-infected HUH-7.5 cells by RNA sequencing, microarray, and proteomics revealed profound changes in, among others, PPAR signaling and PXR/RXR activation [60]. Viral replication efficiency has been linked to variations in cellular bile salt concentrations using the HCV replicon system [61]. Of note, variants of the human bile salt that export pump *ABCB11* might be associated with sustained virological response [62] and progression towards liver cirrhosis [63]. *In vitro* experiments using HCV replicon-harboring cells have shown that the impact of bile salts on HCV replication might be through the action of FXR rather than a direct effect of bile salts themselves [64]. FXR antagonization by guggulsterone blocked the bile salt-mediated induction of HCV replication, and guggulsterone alone inhibited basal HCV replication by tenfold [64]. Hence, it seems feasible that HCV uses transcriptional activation via FXR. It would certainly be of interest to analyze the HCV-binding of natural *FXR* variants implicated in ICP [35]. We speculate that the increased susceptibility for cholestatic disease might be counterbalanced by decreased susceptibility to hepatotropic viruses.

 Differential regulation of the pre-C and pregenomic promoters of HBV by members of the NR superfamily (HNF4-alpha and PPAR-gamma) has been known for some time [65]. Ramière et al. [66] were able to show that FXR-RXR-heterodimers bind to two motifs on the HBV enhancer II and core promoter regions, which are characterized by high homology to the consensus inverted repeat FXR response elements [66]. The tight connection between hepatotropic viruses, liver nutrition, and metabolism by means of NR is intriguing and might reveal new therapeutic targets or even dietary recommendations to optimize the efficacy of antiviral treatment for HBV or HCV infected patients.

### 3.9. NR and Drug-Induced Liver Injury (DILI)

While diet-induced metabolic overload, alcohol and HCV are the most common insults to the liver, drug-induced injury (DILI) is gaining in prominence due to increasing age and multimorbidity of the general population. The likelihood of medication-induced liver damage increases substantially beyond the threshold of 50 mg per day cumulative ingestion [67]. The involvement of the bile salt transporter system in estrogen-induced cholestasis has been observed for a long time in patients with ICP [35, 68, 69]. Functional variants of *ABCB11* are known to be associated with cholestasis induced by oral contraceptives and other drugs [70], as reviewed elsewhere [71].

 The role of gene polymorphisms in predisposition towards DILI has been reviewed comprehensively [72, 73]. Suffice to say that next to drug-metabolising enzymes, drug transporters and genes for the immune response to injury, variants in the NR genes *PXR* [74] and *CAR* [58, 75, 76] are the most prominent contributors, for example, towards acetaminophen (APAP) toxicity. Evidence comes from knockout or knockdown of these genes, conferring resistance to the toxic effects of APAP.

## 4. The Search for New Targets

### 4.1. NR and Metabolic Traits

Identifying and quantifying genes associated with metabolic traits is one of the prime challenges when devising means to deal with the epidemics of diabetes and metabolic syndrome. This information might be used as cost-efficient leverage to identify and motivate at-risk individuals towards lifestyle changes, but to date there is no evidence for clinical impact [77]. A long standing question with regard to the usefulness of genome wide association studies (GWAS) has been how to detect the impact of other than high-frequency low-risk variants. While the setup of GWAS is a case-control scenario in an otherwise unselected population, more in-depth information is expected from longitudinal studies on large cohorts of individuals with defined but limited genetic heterogeneity. A combination of results from both approaches might be required: since GWAS needs to accommodate higher levels of heterogeneity, and hence a greater variety of factors that influence the trait under examination, only factors that reach the threshold across the total population are identified. Studies on smaller cohorts, possibly with less genetic variety due to a relatively small common founder population, enable researchers to identify the impact of comparatively rare variants that have been enriched in the respective population due to a lack of admixture, while contributory genetic factors that are not present in the founder population might be missed. In fact, using large cohorts with relatively small founder populations have enabled researchers to identify metabolic risk factors by GWAS and association mapping. Typing 4,763 individuals from the Northern Finland birth cohort 1966 for 329,091 SNPs identified 21 genomic loci that were associated with metabolic traits such as HDL and LDL cholesterol levels [78]. Besides the “usual suspects” such as a frequent polymorphism in the *NR1H3* gene encoding LXR-alpha, which affects HDL cholesterol level with an effect size of only 4% but shows a risk allele frequency of 42%, the study also detected some higher impact rare alleles. A variation in the gene for the androgen receptor AR (*NR3C4*) exerted a strong influence on LDL cholesterol levels, with a considerable effect size of 30% set aside a risk allele frequency of only 2% [78] (Table 1).

### 4.2. Complex NR Genetics

At our present state of knowledge, the variation of transcript abundance is probably a more important mechanism underlying disease susceptibility than structural protein alterations by nonsynonymous SNPs [79]. The view of health as a balanced state or equilibrium of interrelated gene expressions holds a lot of attraction for liver homeostasis, with diseases representing “network perturbations” [80]. This view also explains why transcription factors are more likely to be detected in GWAS studies of quantitative metabolic traits, since they might be master regulators of multiple other genes. Hence, NR variants impact not just on their own expression but can rather, via multiple effectors, exert stronger effects on a complex phenotype.

 A GWAS with more than 2.5 million SNPs in 19,840 individuals, with replication in up to 20,632 individuals, identified more than 30 loci that had an impact on serum LDL cholesterol, HDL cholesterol, or triglyceride levels. A rare variant (risk allele frequency 3%) in *HNF4A *(p.T130I) was identified as a new contributory factor for HDL cholesterol levels, with an effect size of 19% [81].

### 4.3. Metabolic Traits as Quantitative Trait Loci (QTLs)

Technical progress, particularly the combination of increased speed and decreased cost of genotyping and expression quantification, is ushering in systematic approaches to metabolic traits. Once sufficiently large numbers of individuals from any population (humans, animals, or plants) have been genotyped for markers covering the entire genome, statistics can be used to calculate the likelihood of correlation between the inheritance of genotypes and the expression values of quantifiable phenotypes, including the abundance of transcripts assayed by expression arrays. This methodology, denoted as “expression quantitative trait loci (eQTL) mapping” can be used to unravel gene regulation by association and identify regulatory networks involving multiple loci. To extend the information from these large datasets, Schadt et al. [82] proposed mathematical models and tools to infer relationships between genes and groups of genes as well as between gene expression and disease phenotypes. Liu et al. [83] used data from the mouse phenome database covering 173 mouse phenotypes to map 937 quantitative trait loci *in silico*. Phenotypes examined included various metabolic traits such as fat mass at different diets, cholesterol, and triglyceride levels under normal and atherogenic diet. Ten of the QTL regions identified in this study contained candidate genes that had previously been characterized and shown to cause metabolic phenotypes in agreement with the trait used for mapping, serving as a proof of principle for the application of this methodology.

 A note of caution is obviously due when transferring data and results from mouse models into the human genomic context. Transcriptional regulation, particularly organ-specific mechanisms and binding sites, has diverged significantly between man and mouse, probably more so than NR function [84]. Nevertheless, results from animal models, particularly knockouts, still provide valuable clues towards unexpected mechanisms of disease [85, 86].

### 4.4. Combining the Power of GWAS and eQTL

The identification and functional characterization of regulatory SNPs have encouraged the use of eQTL data for the interpretation of GWAS results. These genome-wide scans using anonymous SNP markers usually detect associations of disease with polymorphic markers, often in regions without known candidate genes [87]. For an example we refer to Kathiresan et al. [81], who identified 30 loci that contributed towards the regulation of three dyslipidemia traits. Increasing knowledge about *trans*regulatory effects by eQTL might help to pinpoint functional mechanisms for these disease-associated variants. eQTL data enables us to identify a mechanism by which a SNP controls expression of a remote locus, hence causing predisposition to disease by allelic variation across long genomic distances [88].

## 5. Bringing It Together: Future Perspectives

Technical advances increase our knowledge regarding the biochemical and gene-regulatory mechanisms underlying metabolic diseases. Transgenic animals inform us about gene function, eQTL studies in human samples and model systems provide us with information about genetic loci that are associated with the inheritance of multiple metabolic parameters, and GWAS in genetically well-characterized cohorts yield candidate genes for metabolic disturbances at ever increasing resolution and depth. Considering all this amassed knowledge, our understanding of disease pathobiology is improving constantly. Considering the reality of how little lifestyle modification is achieved by risk information, as illustrated by antismoking campaigns, it remains a challenging task to employ this knowledge to combat the epidemics of the metabolic syndrome and its associated burden of disease.

## Figures and Tables

**Table 1:** Single nucleotide polymorphisms (SNPs) associated with liver disease.

Gene	SNP	*rs number*	Disease	*OR* (95% CI)	*P*-value	Cohort (controls)	Population	Reference
HNF4a (NR2A1)	Q268X	*rs6093980*	MODY-1	N/A	N/A	>360	R-W pedigree	[6]
	Y16X	N/A		N/A	N/A	108	UK	[9]
	S34X							
	R127W							
	D206Y							
	E276Q							
	R303H I314F L332P							
	M364R							
	c. IVS5nt+1G>A							
	c.IVS4nt-2A>G							
	T(3;20)							
	V393I	N/A	NIDDM	N/A	N/A	N/A	F-40 pedigree	[8]
	T130I	N/A	T2D	1.26 (1.01–1.57)	0.04	1,466 (4,520)	Danish	[10]
	V255M	N/A	Decreasing fasting serum C-peptide levels	1.0 (0.28–3.65)	1.0			

FXR (NR1H4)	−20,647T>G	N/A	Gallstones	0.42 (0.17–1.01)	0.053	77 (74)	Mexican	[34]
	−1G>T	*rs56163822*		0.25 (0.07–0.95)	0.042	75 (70)		
	IVS7-31A>T	*rs7138843*		0.47 (0.22–1.01)	0.053	77 (88)		
	−1G>T	*rs56163822*	ICP	0.92 (0.35–2.44)	0.96	342 (349)	British/Swedish	[35]
	M173T	N/A		3.2 (1.1–11.2)	0.02			

VDR (NR1I1)	c.1025-49G>T (ApaI)	*rs7975232*	AIH	0.72 (0.40–1.30)	0.27	123 (214)	Caucasian	[22]
	Intron 8 (BsmI)	*rs1544410*		0.63 (0.37–1.06)	0.08			
	Exon 2 (FokI)	*rs1073581*		0.5 (0.28–0.92)	0.02			
	I352I (TaqI)	*rs731236*		1.27 (0.69–2.33)	0.43			
	c.1025-49G>T (ApaI)	*rs7975232*	PBC	1.85 (1.02–3.35)	0.04	74 (214)		
	Intron 8 (BsmI)	*rs1544410*		2.1 (1.22–3.62)	0.006			
	Exon 2 (FokI)	*rs1073581*		0.55 (0.27–1.12)	0.09			
	I352I (TaqI)	*rs731236*		1.16 (0.56–2.39)	0.69			
	c.1025-49G>T (ApaI)	*rs7975232*	AIH	0.82 (0.42–1.58)	0,55	49	Chinese	[20]
	Intron 8 (BsmI)	*rs1544410*		1.44 (0.59–3.51)	0.42			
	Exon 2 (FokI)	*rs1073581*		2.18 (1.07–4.43)	0.019			
	I352I (TaqI)	*rs731236*		0.00 (0.00)	0.28			
	c.1025-49G>T (ApaI)	*rs7975232*	PBC	0.90 (0.49–1.64)	0.727	58		
	Intron 8 (BsmI)	*rs1544410*		4.41 (1.29–15.02)	0.01			
	Exon 2 (FokI)	*rs1073581*		1.30 (0.63–2.68)	0.05			
	I352I (TaqI)	*rs731236*		0.00 (0.00)	0.224			
	c.1025-49G>T (ApaI)	*rs7975232*	PBC	0.71 (0.47–1.08)	0.133	195 (179)	Japanese	[21]
	Intron 8 (BsmI)	*rs1544410*		0.71 (0.44–1.16)	0.179			
	I352I (TaqI)	*rs731236*		1.02 (1.00–1.04)	0.109			
	c.1025-49G>T (ApaI)	*rs7975232*		1.02 (0.52–1.98)	1.000	139 (156)	Italian	
	Intron 8 (BsmI)	*rs1544410*		0.33 (0.12–0.92)	0.039			
	I352I (TaqI)	*rs731236*		0.94 (0.51–1.75)	0.876			
	c.1025-49G>T (ApaI)	*rs7975232*	HBV	3.3 (1–11)	0.05	214 (408)		[23]
	c.1025-49G>T (ApaI)	*rs7975232*	HCC	0.852 (0.345–2.113)	n.s.	80 (160)	Caucasian	[30]
	Intron 8 (BsmI)	*rs1544410*		1.711 (0.766–3.813)	n.s.			
	Exon 2 (FokI)	*rs1073581*		1.338 (0.605–2.968)	n.s.			
	I352I (TaqI)	*rs731236*		0.491 (0.212–1.141)	0.09			

PPAR*γ* (NR1C3)	P12A	*rs1805192*	T2D	0.78 (0.59–1.05)	0.045	333	Scandinavian	[40]
				1.37	0.04	2,126 (1,124)	French	[38]
				0.12 (0.03–0.52)	0.005	532 (386)	Asian Sikh	[39]
	C161T	*rs121909245*	Obesity	2.33 (1.03–5.29)	0.042	292 (371)	Australian	[37]
			NAFLD	4.606 (3.744–10.263)	0.003	96 (96)	Chinese	[46]

LXR*α* (NR1H3)	N/A	*rs2167079*	HDL cholesterol level	N/A	5.13 × 10^−8^	4763	Northern Finland Birth cohort 1966	[78]
		*rs7120118*			3.57 × 10^−8^			
AR (NR3C4)	N/A	*rs5031002*	LDL cholesterol level	N/A	2.37 × 10^−7^			

PXR (NR1I2)	Intronic	*rs7643645*	NAFLD	3.48 (1.25–10.62)	0.008	188	Argentine	[54]
		*rs2461823*		N/A	0.039			
	−25385	*rs3814055*	DILI	3.37 (1.55–7.30)	0.0023	51 (64)	European	[74]

Abbreviations: OR: odds ratio; N/A: not annotated; n.s.: not significant.
